# Prevention of Colorectal Cancer by Targeting Obesity-Related Disorders and Inflammation

**DOI:** 10.3390/ijms18050908

**Published:** 2017-04-26

**Authors:** Yohei Shirakami, Masaya Ohnishi, Hiroyasu Sakai, Takuji Tanaka, Masahito Shimizu

**Affiliations:** 1 Department of Informative Clinical Medicine, Gifu University Graduate School of Medicine, Gifu 501-1194, Japan; 2 Department of Gastroenterology, Gifu University Graduate School of Medicine, Gifu 501-1194, Japan; om19840905@gmail.com (M.O.); sakaih03@gifu-u.ac.jp (H.S.); shimim-gif@umin.ac.jp (M.S.); 3 Department of Pathological Diagnosis, Gifu Municipal Hospital, Gifu 500-8513, Japan; tmntt08@gmail.com

**Keywords:** colorectal cancer, chemoprevention, inflammation, obesity, green tea

## Abstract

Colorectal cancer is a major healthcare concern worldwide. Many experimental and clinical studies have been conducted to date to discover agents that help in the prevention of this disease. Chronic inflammation in colonic mucosa and obesity, and its related metabolic abnormalities, are considered to increase the risk of colorectal cancer. Therefore, treatments targeting these factors might be a promising strategy to prevent the development of colorectal cancer. Among a number of functional foods, various phytochemicals, including tea catechins, which have anti-inflammatory and anti-obesity properties, and medicinal agents that ameliorate metabolic disorders, might also be beneficial in the prevention of colorectal cancer. In this review article, we summarize the strategies for preventing colorectal cancer by targeting obesity-related disorders and inflammation through nutraceutical and pharmaceutical approaches, and discuss the mechanisms of several phytochemicals and medicinal drugs used in basic and clinical research, especially focusing on the effects of green tea catechins.

## 1. Introduction

Colorectal cancer (CRC) is considered as a heterogeneous disease characterized by multiple genetic mutations and epigenetic alterations in genes that regulate cell growth and differentiation [1]. In most cases, CRC is non-hereditary (sporadic) because of the sequential accumulation of mutations in multiple genes. Numerous molecular genetic studies have identified several essential gene defects associated with sporadic CRC [2]. CRC is known to be a common malignant disease with a high mortality rate, and its clinical incidence has increased gradually over the past decade [3]. Therefore, more attention should be focused on the prevention and screening methods in patients with a high risk of CRC.

Chronic inflammation is a key predisposing factor to CRC development [4], which is one of the major complications in inflammatory bowel disease (IBD), including ulcerative colitis and Crohn’s disease [5,6]. Obesity is considered an important health issue, and has become more prevalent in the recent years worldwide [7]. Recent epidemiological and experimental evidence has indicated that obesity and related metabolic abnormalities, especially diabetes mellitus, are associated with the development of various malignancies, including CRC [8,9]. Several pathophysiological mechanisms of interaction between obesity and CRC have been studied, including insulin resistance, adipocytokine imbalances, alterations in the insulin-like growth factor (IGF)-1/IGF-1 receptor (IGF-1R) axis, chronic inflammation, and oxidative stress [8,9,10,11,12]. These studies suggest that targeting the pathophysiological interactions using nutritional and/or pharmaceutical interventions could be a promising strategy to prevent colorectal tumorigenesis.

Numerous studies have reported the beneficial effects of green tea catechins (GTCs) on improving the metabolic abnormalities such as obesity, thus preventing the development of malignancies [13,14,15,16]. Another plant-derived substance, curcumin, which is a component of turmeric, and a form of carotenoid, astaxanthin, have also been demonstrated to have preventive effects against colorectal carcinogenesis [17,18,19,20,21]. Branched-chain amino acid (BCAA) supplements, containing essential amino acids such as leucine, isoleucine, and valine could alleviate protein malnutrition and exert anti-cancer properties by ameliorating insulin resistance [22]. Pharmaceutical approaches using the 3-hydroxy-3-methylglutaryl coenzyme A (HMG-CoA) reductase inhibitor pitavastatin, anti-hypertensive drugs, histamine receptor antagonists, and an anti-inflammatory agent pentoxifylline have been investigated and reported to attenuate chronic inflammation and reduce oxidative stress, leading to the prevention of colonic neoplastic lesion development [17,20,23,24,25,26,27,28]. 

The current review summarizes the most promising strategies for the prevention of CRC by targeting obesity-related disorders and inflammation through nutritional and/or pharmaceutical approaches with several of the phytochemicals and medicinal drugs described above, because these agents have been closely studied in obesity-associated CRC models. In addition, this review article also further discusses the mechanisms of several phytochemical, especially GTCs, and medicinal agents (used in basic and clinical research) responsible for the chemoprevention of CRC. 

## 2. Preventive Effects of Green Tea and Its Constituents on CRC Development

Several population-based studies have indicated that the consumption of green tea provides protective effects against CRC development [29,30,31]. A prospective cohort study investigating the effects of green tea intake on CRC incidence and mortality has demonstrated that green tea consumption lowers the risk of CRC-related mortality with a moderate dose-response relationship [32]. A meta-analysis study discussing the association between green tea intake and the risk of CRC development reported several case-control studies showing an inverse correlation between green tea consumption and CRC risk, while many other studies reported no correlation [33].

**Figure 1:**
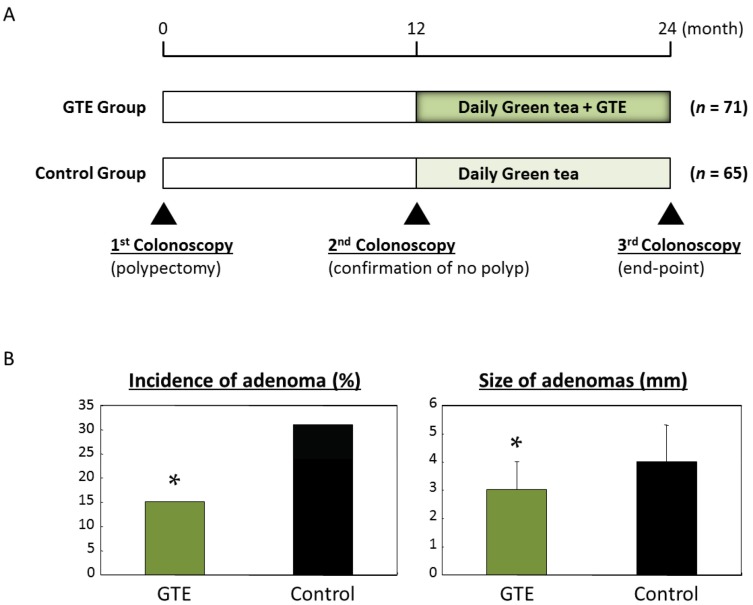
Protocol of a pilot study to investigate chemopreventive effects of green tea extracts on metachronous adenomas in the colorectum after polypectomy. (**A**) The study included 136 participants who underwent endoscopic resection of colorectal adenomas. In 12 months, the participants received a second colonoscopy to confirm the absence of detectable adenoma. The participants were then randomized into two groups: the GTE group (*n* = 71) was given three green tea extracts (GTEs) tablets per day for 12 months and the control group (*n* = 65) received no supplementation; (**B**) After 12 months of GTE administration, the end-point colonoscopy was performed in 125 patients to check for the presence of new colonic adenomas. Administration of 1.5 g/day of GTEs for 12 months successfully inhibits the development of colorectal adenoma compared to the control group. * *p* < 0.05.

Only a few interventional clinical trials have examined the chemopreventive effects of green tea on CRC development. In a pilot study, we investigated the effects of green tea extracts (GTEs) on the development of colorectal adenoma, a pre-cancerous lesion in the colorectum [34]. Patients who had undergone polypectomy for the removal of colorectal adenomas participated in the trial (Figure 1A). We have found that the administration of 1.5 g of GTEs per day for one year successfully inhibited the development of metachronous colorectal adenoma in comparison with the control group (Figure 1B). The study also demonstrated that the size of recurrent adenomas in the GTE-administered group was significantly smaller than that of the untreated control group, and no adverse events were observed in the treatment group.

The anti-cancer activity of green tea and its constituents has been demonstrated by in vitro studies and in chemically- or genetically-induced animal models of various tumors, including the lungs, skin, esophagus, stomach, liver, pancreas, bladder, small and large intestines, and prostate [35,36,37]. A number of studies have also investigated the effects of green tea and its constituents on CRC development. Chen et al. [38] have reported that the treatment of human colon cancer cells with (–)-epigallocatechin-3-gallate (EGCG), a tea catechin and a major biologically active component in green tea, inhibits the growth of the cancer cells. Our research group has shown that both EGCG and standardized polyphenol polyphenon E (PolyE), which contains 65% EGCG, 25% other catechins, and 0.6% caffeine, can preferentially inhibit the growth of various human colon cancer cells [39]. We have also found that the growth of human CRC xenografts was markedly reduced by the administration of EGCG [40]. Another in vivo experiment using a chemically induced rat CRC model has demonstrated that the consumption of green tea significantly suppresses the development of premalignant aberrant crypt foci (ACF) lesions in the colorectum [41].

**Figure 2:**
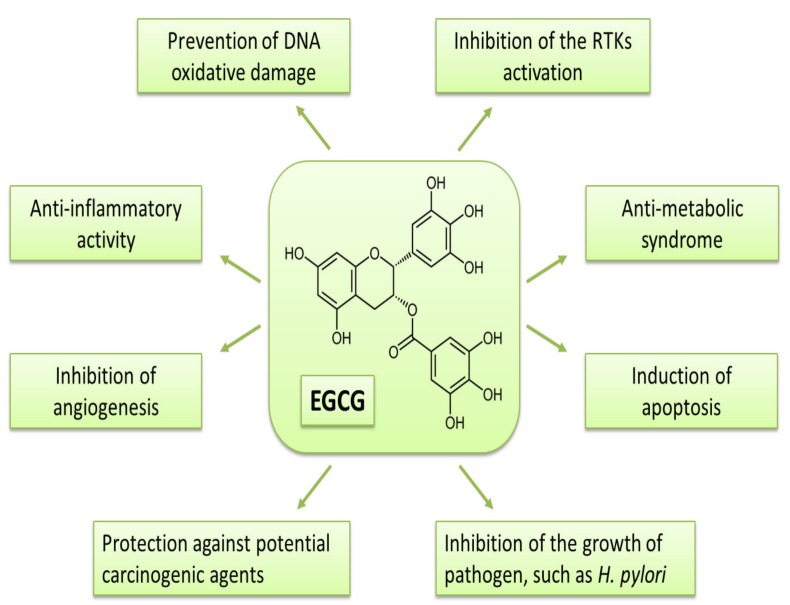
Proposed mechanisms of action of EGCG against malignancy.

Previous studies have demonstrated that receptor tyrosine kinases (RTKs) are one of the important targets of EGCG to inhibit cancer cell growth. EGCG inhibits the activation of subclass I proteins of the RTK superfamily, including EGFR, HER2, and HER3, in various cancer cells [39,42]. Activities of other RTK superfamily proteins, such as IGF-1R and vascular endothelial growth factor (VEGF) receptors, are also shown to be inhibited by EGCG. Hence, the RTK-associated cell signaling, such as the Ras/MAPK and PI3K/Akt pathways, is thought to be down-regulated in cancer cells by EGCG, leading to the modulation of the target gene expression, which is associated with the induction of apoptosis and cell cycle arrest. The molecular mechanisms which explain how EGCG affects RTK signaling have been studied in detail by Adachi et al. [43,44,45]. The studies indicate a target of EGCG for anti-cancer mechanisms associated with RTKs, particularly detergent-insoluble ordered plasma membrane domains “lipid rafts”, which are important as signal processing hubs of RTKs. EGCG alters the lipid organization on the plasma membrane and induces the EGFR internalization of endosomes, which prevents ligands from binding to receptors. The degradation of EGFR due to internalization appeared to be induced by phosphorylation of the receptor, which is associated with the activation of p38 MAPK by EGCG. This suggested mechanism may be able to explain the ubiquitous effects of EGCG on various types of RTKs, because most RTKs function on lipid rafts. Among RTKs, IGF-1R is thought to be one of the most critical targets for the inhibition of obesity-related carcinogenesis by tea catechins, although the direct alteration of catechins on IGF-1R needs to be clarified. For more details on the effects of EGCG on RTKs and other anti-neoplastic efficacy, please refer to the review articles by Shimizu et al. [13,14] and to Figure 2, which summarizes the properties.

Chronic inflammation plays a vital role in carcinogenesis, including CRC [4], which is known as one of the most serious complications of IBD [5,6]. Persistent inflammation, characterized by the production of pro-inflammatory cytokines, causes oxidative damage to DNA, mutations in oncogenes and tumor suppressor genes, including adenomatous polyposis coli (APC), p53, and K-ras, and genomic instability, leading to colitis-associated tumor development. While it is considered that inflammation does not initiate sporadic CRC, chronic inflammation is also known to facilitate tumor promotion, progression, and metastasis in the pathogenesis of colitis-associated and sporadic CRC [46]. Tanaka et al. [47] have developed an experimental mouse model of inflammation-related colon carcinogenesis induced by the administration of azoxymethane (AOM) and dextran sodium sulfate, which mimics the chronic intestinal inflammation that occurs in IBD. Employing this rodent model, we demonstrated the suppressive effects of EGCG and PolyE on inflammation-related colon carcinogenesis [48]. In this study, EGCG or Poly E significantly suppressed the multiplicity and volume of colonic neoplasms. In addition, treatment with EGCG or Poly E decreased the protein and mRNA expression levels of cyclooxygenase (COX)-2 and the mRNA expression of inflammatory cytokines, including tumor necrosis factor (TNF)-α, interferon-γ, interleukin (IL)-6, IL-12, and IL-18 in the colonic mucosa. Previous studies have indicated that EGCG or green tea extract reduces the expression of TNF-α and IL-6 via attenuating NF-κB activity [49]. These results suggest that tea catechins can ameliorate colonic inflammation and have beneficial effects for inhibiting the development of cancer in the inflamed colon. 

Recent epidemiological and experimental evidence has indicated that obesity is related to the incidence of CRC [8,9,10,50]. Insulin resistance and hyperinsulinemia, metabolic disorders associated with obesity, are considered important risk factors for CRC development [51]. It is reported that insulin and its regulated signal transduction network play important roles in carcinogenesis [52,53,54]. Many studies have shown that the IGF-1/IGF-1R axis plays a key role in the carcinogenesis of various cancers, including CRC [52,53,54]. In addition, insulin resistance and an increased fat mass induce oxidative stress in tissues and increase the expression of various pro-inflammatory cytokines, including TNF-α and IL-6, which further lead to the growth and progression of malignancies [55,56,57]. Oxidative stress induces DNA damage and activates the PI3K/Akt signaling pathway, both of which are thought to promote cancer development [58,59]. Therefore, insulin resistance, inflammation, and oxidative stress can be considered as important factors in the development of obesity-related CRC [60,61]. This imbalance is usually caused by enhanced fat storage, increased levels of leptin, and decreased levels of adiponectin in the serum [60,61]. Leptin induces the production of TNF-α and IL-6 [62,63], and thus stimulates CRC cell growth [64]. Moreover, an epidemiological study has reported a positive correlation between the circulating leptin levels and CRC development [65]. These findings suggest that obesity-associated abnormalities cooperatively increase the risk of CRC in obese individuals.

A genetically-modified C57BLKS/J-+Lepr^db^/+Lepr^db^ (db/db) mouse exhibiting the characteristics of obesity and type 2 diabetes was recognized as a useful model for investigating various types metabolic disorders [66]. Hirose et al. [67] have shown that db/db mice have hyperlipidemia, hyperinsulinemia, and hyperleptinemia, and are susceptible to the colonic carcinogen AOM. We used a db/db mouse and investigated the effects of EGCG on AOM-induced colon carcinogenesis [68], and observed that EGCG markedly decreases the total number of ACF and β-catenin accumulated crypts (BCACs), both of which are premalignant lesions in the colorectum. Additionally, we found decreased IGF-1 and restored IGF binding protein-3 (IGFBP-3) levels in serum and down-regulated levels of COX-2, cyclin D1, and the activated form of IGF-1R in colonic mucosa upon EGCG administration. With regard to the IGF/IGF-1R axis, treatment with EGCG showed decreased levels of IGF-1 and reduced IGF-1R activation, whereas the levels of IGFBP-3 were found to be increased in colon cancer cells [69].

## 3. Prevention of CRC through a Nutraceutical Approach

The colorectal mucosa of a db/db mouse expresses higher levels of the activated form of IGF-1R, β-catenin, and COX-2 than the control [67]. In accordance with the study demonstrating the effects of EGCG on AOM-induced colon premalignant lesions in db/db mice [68], dietary supplementation with other types of phytochemicals was also found to suppress the development of pre-cancerous lesions in the db/db mice [70,71]. In addition, we have used this rodent model to investigate the chemopreventive effects of curcumin, a yellow pigment in the rhizome of the spice turmeric with known anti-inflammatory properties [72,73], on obesity-related carcinogenesis. Kubota et al. [18] have demonstrated that the administration of curcumin successfully prevents the development of colonic premalignant lesions in AOM-injected db/db mice by inhibiting the NF-κB activity and down-regulating the expression of TNF-α, IL-6, and COX-2, further ameliorating the adipokine imbalance. Moreover, a type of carotenoid, astaxanthin, inhibited the development of colonic premalignant lesions in the same carcinogenesis model by reducing leptin levels, inhibiting NF-κB activation, and attenuating chronic inflammation and oxidative stress in the colonic mucosa [17]. Furthermore, supplementation with amino acid-preparation BCAA caused a significant decrease in the number of ACF and BCAC in the same colon tumorigenesis model [22]. The test group administered with BCAA demonstrated reduced levels of COX-2, cyclin D1, Akt, and the activated form of IGF-1R in mucosa and decreased serum levels of insulin, IGF-1, IGF-2, triglycerides, total cholesterol, and leptin [22].

These observations suggest that supplementation with certain kinds of phytochemicals and carotenoids or BCAA effectively suppresses the development of premalignant lesions of CRC by attenuating chronic inflammation, down-regulating the IGF/IGF-1R axis, improving dyslipidemia, ameliorating hyperleptinemia, and/or inhibiting the expression of COX-2, which appears to be a promising target for the prevention of CRC [74,75].

## 4. Prevention of CRC through a Pharmaceutical Approach

There are several reports of clinical trials examining the effects of non-steroidal anti-inflammatory drugs such as celecoxib, aspirin, and metformin on the development of CRC or its precursor lesion adenomatous polyp in patients, where these agents appear to be promising [76,77,78,79,80]. The recent randomized and placebo-controlled clinical trials are summarized in Table 1. Recently, we reported that pentoxifylline, which is a methylxanthine derivative and known to possess anti-inflammatory effects, attenuated chronic inflammation and oxidative stress, leading to the prevention of colonic tumorigenesis in an obesity-related colon cancer model [23]. In addition, our research group also demonstrated that histamine and histamine receptors appeared to be critical molecules during inflammation and carcinogenesis in the colorectum, and that several histamine receptor antagonists might be potential chemopreventive agents for inflammation-related CRC development [26].

Several studies have indicated the anti-cancer properties of drugs related to metabolic disorders. Statins and HMG-CoA reductase inhibitors are widely recognized as effective agents against dyslipidemia. In addition, statins have been shown to possess anti-cancer properties [81]. Statins induce apoptosis in CRC cells, attenuate colonic inflammation, and suppress inflammation-related colorectal carcinogenesis in mice [28,82]. Several epidemiological studies have also demonstrated the chemopreventive effects of statins on various malignant diseases, including CRC [81,83]. In our previous study, we demonstrated the cancer preventive effects of a lipophilic statin, pitavastatin, on AOM-induced colorectal carcinogenesis in a db/db mouse model [27]. We found that pitavastatin administration significantly reduced the number of pre-neoplastic BCAC lesions, which may have been caused by the inhibition of the proliferation and decrease in the expression levels of COX-2 and pro-inflammatory cytokines, such as TNF-α and IL-6, in the colonic mucosa. Pitavastatin also elevated the serum levels of adiponectin, while reducing the serum levels of leptin, TNF-α, and IL-6 [27].

Hypertension and dyslipidemia are thought to be involved in obesity-related diseases [9,84]. The activation of the renin-angiotensin system (RAS) has been shown to contribute to high blood pressure, obesity, and metabolic syndrome [85]. RAS has been demonstrated to be frequently up-regulated in malignancies attributed to systemic oxidative stress and hypoxia, which are thought to trigger a state of chronic inflammation [86]. We investigated the effects of anti-hypertensive agents on the prevention of colorectal premalignant lesions in an obesity-related CRC model [25]. The employed agents were an angiotensin-converting enzyme inhibitor, captopril, and an angiotensin-II type 1 receptor blocker, telmisartan, both of which have the ability to inhibit the RAS, and are widely used in clinical practice. The development of colorectal lesions, ACF and BCAC, was significantly inhibited by the treatment with either captopril or telmisartan. These agents markedly decreased the expression levels of TNF-α in the colonic mucosa, and also reduced oxidative stress in the body [25]. Captopril was also reported to prevent the development of ACF by a similar mechanism in diabetic and hypertensive rats [24].

The findings discussed above suggest that both lipid-lowering and anti-hypertensive agents can suppress obesity-associated colorectal carcinogenesis by improving hyperleptinemia and dyslipidemia, and by attenuating chronic inflammation in the colorectum. Therefore, the pharmaceutical approach appears to be one of the potential strategies for the prevention of obesity-related CRC because these drugs are in clinical use and have known pharmacological effects against the obesity-related metabolic disorders, in addition to their cancer chemopreventive effects.

## 5. Concluding Remarks

**Figure 3:**
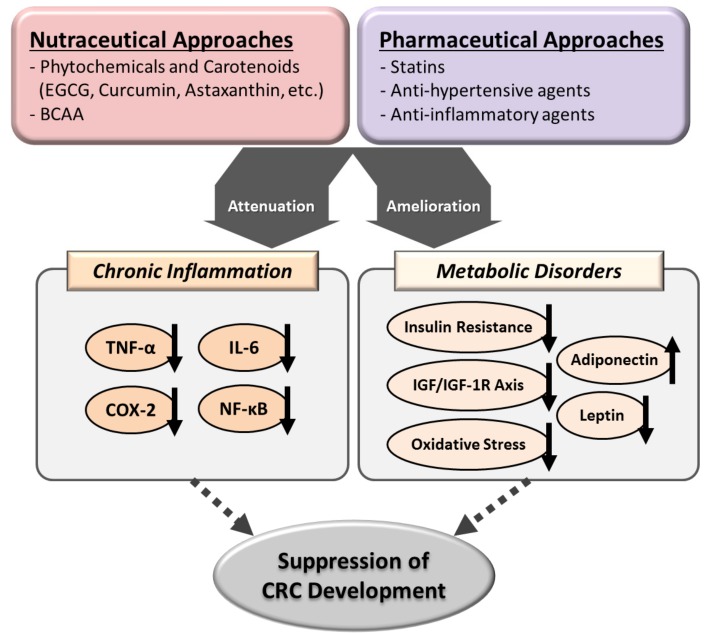
Proposed mechanisms of action of several nutraceuticals and pharmaceuticals in the suppression of colorectal carcinogenesis. The upward and downward arrows indicate up-regulation and down-regulation, respectively.

In this review article, we have discussed the use of nutraceutical and pharmaceutical approaches as promising strategies to prevent CRC development by targeting chronic inflammation and ameliorating metabolic disorders (Figure 3). Moreover, GTCs are easily available and are considered safe based on the long history of their global use. Several interventional studies on humans have also demonstrated that the consumption of GTCs, even in relatively high doses, has no serious adverse reactions [34,87,88], while clinical trials have reported that drugs such as celecoxib can increase the risk of cardiovascular events [76]. In addition, BCAA, statins, and anti-hypertensive drugs are widely used and have beneficial effects on various metabolic disorders. Hence, active intervention using these agents may be a promising strategy for the chemoprevention of CRC.

## Figures and Tables

**Table 1:** The recent randomized and placebo-controlled clinical trials using medicinal agents for the prevention of CRC.

Reference	Agent	Target Lesion	No. of Subjects	Observation Period	Preventive Effects
2006 Bertagnolli [71]	Celecoxib (200 or 400 mg twice a day)	Sporadic colorectal adenomas	2035 subjects; placebo (679) or 200 mg (685) or 400 mg (671) of celecoxib group	Either one and three years	The estimated cumulative incidence of adenomas by year 3 was lower in those receiving 200 mg (risk ratio 0.67 [95% CI: 0.59–0.77]) and 400 mg celecoxib (risk ratio 0.55 [95% CI: 0.48–0.64]).
2006 Arber [72]	Celecoxib (400 mg/day)	Sporadic colorectal adenomatous polyps	1561 subjects (628 in the placebo and 933 in the celecoxib group)	Either one and three years	The cumulative rate of adenomas detected through year 3 was lower in the celecoxib group; relative risk 0.64 (95% CI: 0.56–0.75).
2013 Ishikawa [73]	Aspirin (100 mg/day)	Polyps in patients with familial adenomatous polyposis (FAP)	34 subjects with FAP (17 each in the aspirin and placebo groups)	Six-ten months	The increase in mean diameter of polyps tended to be greater in the placebo group compared to the aspirin group.
2014 Ishikawa [74]	Aspirin (100 mg/day)	Colorectal adenomas and adenocarcinomas	311 subjects (159 in the placebo and 152 in the aspirin group)	Two years	The subjects treated with aspirin displayed reduced colorectal tumourigenesis; adjusted OR 0.60 (95% CI: 0.36–0.98).
2016 Higurashi [75]	Metformin (250 mg/day)	Sporadic colorectal polyps	151 subjects (72 in the placebo and 79 in the metformin group)	One year	The prevalence of total polyps and adenomas in the metformin group was significantly lower; (total polyps) risk ratio 0.67 (95% CI: 0.47–0.97), (adenomas) risk ratio 0.60 (95% CI: 0.39–0.92).

## Abbreviations

ACF	aberrant crypt foci
AOM	azoxymethane
BCAA	branched-chain amino acid
BCAC	β-catenin accumulated crypt
CI	confidence interval
CRC	colorectal cancer
EGCG	(–)-epigallocatechin-3-gallate
FAP	familial adenomatous polyposis
GTC	green tea catechin
GTE	green tea extract
HMG-CoA	3-hydroxy-3-methylglutaryl coenzyme A
IBD	inflammatory bowel disease
IGF	insulin like growth factor
OR	odds ratio
PolyE	Polyphenon E
RAS	renin-angiotensin system
RTK	receptor tyrosine kinae
VEGF	vascular endothelial growth factor
